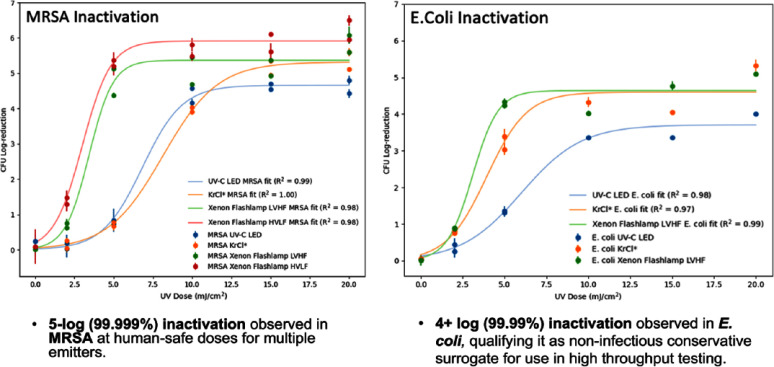# Investigating Microbial Mitigation for Surgical Incision Sites Using UV-C

**DOI:** 10.1017/ash.2025.213

**Published:** 2025-09-24

**Authors:** Ben Robertson, Nancy Havill, Eric Prast, Christopher Jones, Deborah Mosca, Katja Auer, Richard Rasansky, Ernest Blatchley, Karl Linden

**Affiliations:** 1 XCMR Inc.; 2 Yale New Haven Health System; 3 VP, Product Engineering; 4 XCMR

## Abstract

**Background:** Surgical site infections (SSIs) are a serious complication following surgery. The emergence of multidrug-resistant pathogens has diminished the effectiveness of traditional antimicrobials necessitating a new approach to prevention and treatment. We are developing an innovative device (xIP) that uses UV-C to inactivate pathogens in surgical incision sites, mitigating the risk of developing an SSI.

Irradiation in the UV-C range (200-280 nm) is known to inactivate surface and airborne pathogens by damaging nucleic acids. However, there is a limited research on its effectiveness for surgical sites. **Methods:** A Krypton-Chloride Excimer (KrCl*) lamp (λpeak = 222 nm), a pulsed Xenon (PX) emitter (broad spectrum), and a UVC LED (λpeak = 282 nm) were evaluated. Inactivation of E. coli ATCC 29425 and MRSA USA300 was determined by in vitro exposure to UV-C at doses of 0 (control), 2, 5, 10, 15, and 20 mJ/cm2. Dosing was controlled by measuring irradiance (mW/cm2) from each lamp and calculating the time to reach desired exposure levels.

Microbial suspensions of log-phase cultures were pelletized and resuspended in phosphate buffered saline three times and diluted to 107 CFU/mL. After UV exposure, suspensions were plated on an agar substrate using a grid-based method. After incubating for 48 hours at 37°C, remaining viability was determined. **Results:** PX and KrCl* emitters exhibited 5+ log reduction for both microorganisms, while LED showed 4 and 4.5 log reduction against E. coli and MRSA, respectively. PX demonstrated the highest inactivation efficiency (log-reduction per unit dose), followed by KrCl* and LED. **Conclusions:** In-vitro data suggest that surgical sites could be effectively treated in less than a minute with a small hand-held device and in less than 10 seconds with a larger device. Inactivation of MRSA using a superficial wound model in hairless SHKI1-elite mice (Charles River strain code 477) is in progress. In-silico modelling using optical raytracing is in progress to understand the impact of wound and skin micro-environment on the performance of the device. These data will inform ex-vivo testing using porcine or cultured human skin (EpiDerm FT) models to evaluate the performance in different wound types including incisions, abrasions, and burns, as well as the impact of fluids like saline and blood. Development of the xIP device is underway in collaboration with healthcare professionals to produce a product that is effective, fits into current practice, and user friendly. Upon successful completion of a prototype device, clinical efficacy will be explored.

**Figure:**